# Synthesis, Antitubercular Activity and Mechanism of Resistance of Highly Effective Thiacetazone Analogues

**DOI:** 10.1371/journal.pone.0053162

**Published:** 2013-01-03

**Authors:** Geoffrey D. Coxon, Derek Craig, Rosa Milagros Corrales, Emilie Vialla, Laila Gannoun-Zaki, Laurent Kremer

**Affiliations:** 1 Strathclyde Institute of Pharmacy and Biomedical Sciences, University of Strathclyde, Glasgow, United Kingdom; 2 Laboratoire de Dynamique des Interactions Membranaires Normales et Pathologiques, Université de Montpellier 2 et 1, CNRS, UMR 5235, Montpellier, France; 3 INSERM, DIMNP, Montpellier, France; Hopital Raymond Poincare - Universite Versailles St. Quentin, France

## Abstract

Defining the pharmacological target(s) of currently used drugs and developing new analogues with greater potency are both important aspects of the search for agents that are effective against drug-sensitive and drug-resistant *Mycobacterium tuberculosis.* Thiacetazone (TAC) is an anti-tubercular drug that was formerly used in conjunction with isoniazid, but removed from the antitubercular chemotherapeutic arsenal due to toxic side effects. However, several recent studies have linked the mechanisms of action of TAC to mycolic acid metabolism and TAC-derived analogues have shown increased potency against *M. tuberculosis*. To obtain new insights into the molecular mechanisms of TAC resistance, we isolated and analyzed 10 mutants of *M. tuberculosis* that were highly resistant to TAC. One strain was found to be mutated in the methyltransferase MmaA4 at Gly101, consistent with its lack of oxygenated mycolic acids. All remaining strains harbored missense mutations in either HadA (at Cys61) or HadC (at Val85, Lys157 or Thr123), which are components of the β-hydroxyacyl-ACP dehydratase complex that participates in the mycolic acid elongation step. Separately, a library of 31 new TAC analogues was synthesized and evaluated against *M. tuberculosis*. Two of these compounds, **15** and **16**, exhibited minimal inhibitory concentrations 10-fold lower than the parental molecule, and inhibited mycolic acid biosynthesis in a dose-dependent manner. Moreover, overexpression of HadAB HadBC or HadABC in *M. tuberculosis* led to high level resistance to these compounds, demonstrating that their mode of action is similar to that of TAC. In summary, this study uncovered new mutations associated with TAC resistance and also demonstrated that simple structural optimization of the TAC scaffold was possible and may lead to a new generation of TAC-derived drug candidates for the potential treatment of tuberculosis as mycolic acid inhibitors.

## Introduction

Drug-resistant *Mycobacterium tuberculosis* is an increasing threat to global health [1], [2]. New drugs to treat tuberculosis are urgently needed, yet the pace of new drug development has been slow. Few new drug targets have been validated despite considerable advances in our understanding of *M. tuberculosis* biochemistry, metabolism and identification of many essential genes and pathways [3]. While it has become apparent that not all essential metabolic processes represent good drug targets [4], years of drug development efforts have shown that the bacterial cell wall is an excellent target for antibacterials [5], [6], [7]. Several successful antitubercular drugs, including isoniazid (INH) and ethionamide (ETH), inhibit enzymes required for mycolic acid synthesis [5], [8], [9]. Mycolic acids are C_60_-C_90_ branched-chain β-hydroxylated fatty acids that are covalently bound to arabinogalactan-peptidoglycan forming the skeleton of the cell wall [10]. They are also found in the abundant non-covalently associated outer membrane ester glycolipids trehalose monomycolates (TMM) and trehalose dimycolates (TDM) [11] or as free lipids in mycobacterial biofilms [12]. Two major mycolic acid classes based on chemical modifications in the meromycolate can be distinguished: the α-mycolic acids possess two cyclopropane rings, while the oxygenated mycolic acids, such as keto- and methoxy-mycolates possess oxygen functions [10]. Mycolic acids display important characteristics such as permeability to antibiotics and persistence within the infected host [13], [14], [15].

The biosynthetic machinery of mycolates involves type I and type II fatty acid synthases, FAS-I and FAS-II, respectively [10]. FAS-II is composed of four dissociable enzymes that act successively and reiteratively to elongate the growing acylated-acyl carrier protein (ACP). FabH links FAS-I and FAS-II, providing a β-ketoacyl-ACP product with two added carbon atoms which is then reduced by the reductase MabA, followed by a dehydratation step carried out by the set of dehydratases HadABC and then reduction by the enoyl-ACP reductase InhA. The subsequent steps of elongation of the growing acyl-ACP chain with the condensation of a malonyl-ACP unit at each round are performed by the condensases KasA and KasB [16]. Most FAS-II enzymes are unique and essential, thus representing excellent drug targets.

There is a vital need for the discovery and development of new antitubercular drugs that can shorten the treatment of drug-sensitive *M. tuberculosis* and are also active against strains of the organism resistant to the currently used drugs. One way to find new agents is to identify the pharmacological targets of currently used drugs and then develop new therapeutic analogues with greater potency, better pharmacokinetics (PK) and less toxicity. When multi-drug or extensive-drug resistance leads to treatment failure with first-line and even second-line anti-TB drugs, all alternative agents are considered, including thiocarbamide drugs such as ethionamide (ETH) thiacetazone (TAC) or isoxyl (ISO), even though these have often shown unacceptable levels of toxic side-effects. However, once their cellular target(s) and mode of action have been discovered and characterized, one could envisage the development of new, more potent drugs attacking the same target(s), but with reduced side-effects.

Many current antitubercular drugs require some form of cellular activation to unmask reactive groups that will bind to their specific targets. ETH, TAC and ISO are all pro-drugs that must be enzymatically activated by the monooxygenase EthA [17]. Expression of EthA is transcriptionally repressed by EthR [18], [19], so when EthR is overexpressed, it blocks *ethA* transcription, thereby generating ETH resistance. Conversely, in the absence of EthR, EthA is overproduced, leading to hypersusceptibility to the drug. Thus, the drug resistance phenotype in clinical isolates can be caused by the lack of drug activation in the target mycobacteria.

Our previous work demonstrated that in *M. bovis* BCG, *M. chelonae* and *M. marinum*, TAC inhibits the methyltransferases that introduce cyclopropane rings into mycolates [20]. Members of this methyltransferase family of enzymes include PcaA, CmaA2 and MmaA2, which are all important in the pathophysiology of mycobacteria [13], [21], [22]. However, inhibition of these enzymes is unlikely to explain the antitubercular activity of TAC. TAC-resistant BCG mutants lacking keto-mycolates were identified and found to possess mutations within the *mmaA4*, which encodes a methyltransferase required for the synthesis of methoxy- and keto-mycolic acids [23]. In addition, we also found several highly resistant TAC mutants without mutations in either *ethA* or *mmaA4*, suggesting that resistance could be mediated by mutations in additional gene(s) [23]. Recently, it was observed that TAC inhibits mycolic acid biosynthesis in *M. tuberculosis* and *M. kansasii* and that resistance was associated with mutations in the *hadABC* operon [24]. That this suggests that the FAS-II dehydratase complex represents a new player in the resistance to this antitubercular compound is also supported by the recent findings that treating *M. tuberculosis* with TAC results in the accumulation of 3-hydroxy fatty acids, the substrates of the dehydratase [25].

The present study, a continuation to our efforts to decipher the mechanisms of resistance and action of TAC, confirmed these recent results and prompted us to synthesize and evaluate the antitubercular activity of a simple series of new TAC analogues.

## Materials and Methods

### Mycobacterial Strains and Growth Conditions

*M. bovis* BCG strain Pasteur 1173P2 and *M. tuberculosis* mc^2^7000 [26] were all grown either on Middlebrook 7H10 agar enrichment or in Sauton’s broth medium supplemented with oleic acid-dextrose-catalase (OADC) at 37°C. For *M. tuberculosis* mc^2^7000, media were supplemented with 24 µg/ml pantothenic acid. Spontaneous mc^2^7000 mutants, resistant to high levels of either TAC or SRI-224 were selected by diluting and plating mid-log phase cultures at approximately 1 × 10^8^ cells/ml on 7H10 agar medium containing either 2.5, 5 or 10 µg/ml of TAC or SRI-224. Plates were incubated at 37°C up to 4 weeks. Single colonies were picked, grown in Sauton’s medium and maintained in culture without drug.

### DNA Manipulation, Sequencing and Genetic Constructs

Sequence of *mmaA4, ethA, hadA, hadB* and *hadC* genes in the various *M. tuberculosis* resistant mutants was obtained from PCR amplicons of the concerned genomic region using the primers listed in Table 1. PCR was done using the high fidelity polymerase from Euromedex, and the resulting products were purified using the PCR clean-up system (Qiagen) and directly sequenced.

**Table 1:** Oligonucleotides used in this study.

Primer	Sequence (5′-3′)	Purpose
HadA-qRT-F	gggcttgctggctccgttga	qRT-PCR analysis of *hadA*
HadA-qRT-R	gcccgccacgatcggtttct	qRT-PCR analysis of *hadA*
HadC-qRT-F	ccgcgggatgatttggcggt	qRT-PCR analysis of *hadC*
HadC-qRT-R	cggccgcttcctcgctcaaa	qRT-PCR analysis of *hadC*
SigA-RT-F	gcccgaggagctggccaaag	qRT-PCR analysis of *sigA*
SigA-RT-R	ccaagctggctgtcgccctc	qRT-PCR analysis of *sigA*
HadA-F-261	caatggccatggcg ttgagcgcagacatcgttggga (MscI site underlined)	Amplification of *hadABC* for cloning into pMV261
HadC-R-261	ccggaattcttacgcggtcctgatgacctgcccg (EcoRI site underlined)	Amplification of *hadABC* for cloning into pMV261
HadA-F	agttctgcccgaattgcggcaaacaccagg	Amplification of *hadABC* for sequencing
HadC-R	cctcggggttttcccaacatcggcgcgct	Amplification of *hadABC* for sequencing
EthA4-F	ggcagcgaagcctgactggccgcggaggtg	Amplification of *ethA* for sequencing
EthA6-R	tgggcggggtgacattcgttccgggcgatatcg	Amplification of *ethA* for sequencing
mmaA4-F	aggcgttccgaatgggctacatcg	Amplification of *mmaA4* for sequencing
mmaA4-R	cgaagttgcgggtgatggatgg	Amplification of *mmaA4* for sequencing

F and R stand for forward and reverse, respectively.

To generate the pMV261_*hadABC* construct, the *hadABC* gene cluster was PCR amplified using primers HadAF-261 and hadCR-261 using *M. tuberculosis* genomic DNA. The amplicon was then restricted by MscI/EcoRI and ligated into the *E. coli*-mycobacterial shuttle vector pMV261 containing the *hsp60* promoter [27] cut with the same enzymes. Integrity of the construct was verified by nucleotide sequencing. The pMK1_*hadAB* and pVV16_*hadBC* constructs allowing to produce in mycobacteria the N-terminal and C-terminal His-tagged heterodimers HadAB and HadBC, respectively, were reported previously [28]. Constructs allowing overexpression HadAB, HadBC or HadABC we used to transform *M. tuberculosis* mc^2^7000, and transformants were selected on 7H10 agar containing OADC, 25 µg/ml kanamycin and 24 µg/ml pantothenate. Plates were incubated at 37°C for 2–3 weeks.

### RNA Extraction and Quantitative Real Time-PCR

5 ml cultures at A_600_ of 0.8-1 were harvested, immediately resuspended in 1 ml of RNA protect reagent (Qiagen), incubated for 1 hr at room temperature and finally resuspended in 1 ml of RLT buffer from the RNeasy kit (Qiagen). Subsequently, bacteria were transferred to a Lysing matrix B tube (MP Bio), processed in a bead beater apparatus for 45 s at maximal speed three times, and held on ice before centrifugation. Supernatants (750 µl) were mixed with 525 µl of ethanol, and RNA was purified with the RNeasy kit according to the manufacturer's instructions (Qiagen). Contaminating DNA was removed following DNase I treatment (Invitrogen). RNA integrity was analyzed on a BioAnalyzer 2100 machine from Agilent, using RNA nano series II chips. Double-stranded cDNA was produced using the Superscript III reverse transcriptase (Invitrogen) as described by the manufacturer.

Quantitative PCR were performed using an in-house SYBR Green mixture and Lightcycler 480 II (Roche Applied Science), as described previously [29]. The PCR program consisted of initial denaturation at 98°C for 3 min, 45 cycles of 98°C for 5 s, 68°C for 10 s and 72°C for 10 s. *HadAB* and *HadBC* genes were amplified with specific primers (Table 1). Crossing point values, given as cycle numbers, were normalized to values obtained for the mycobacterial household gene *sigA.* Primer efficiencies, determined as described previously [29], were between 1.9 and 2.

### Mycolic Acid Analysis

Extraction of cell wall mycolic acids from mycobacterial cells was carried out as previously described [17]. Briefly, cell pellets were washed and treated with 15% tetrabutyl ammonium hydroxide (TBAH) at 100°C overnight. Mycolic acids were methyl-esterified and extracted in diethyl ether. Extracts were dried and resuspended in dichloromethane for application to a silica-coated plate for thin layer chromatography (TLC). Mycolates were resolved on normal phase TLC in hexane/ethyl acetate (19/1, v/v). Lipids were visualized by spraying the TLC plate with 5% molybdophosphoric acid (MPA) in ethanol followed by charring with a heat gun.

For visualizing drug-induced changes in the mycolates profile, increasing drug concentrations of TAC or related analogues were added to the growth medium for 14 hours. Metabolic labeling of lipids was performed by adding 1 µCi/ml of [2-^14^C]acetate (56 mCi/mmol, Amersham Biosciences) for an additional 6 hours at 37°C, followed by harvesting of the cells and extraction of mycolates as described above. Autoradiograms were obtained by overnight exposure to Kodak Biomax MR film to reveal [^14^C]-labeled lipids. In case of silica plates impregnated with 10% silver nitrate, hexane/ethyl acetate (19∶1, v/v) was used for three runs. Two-dimensional TLC on silver nitrate-impregnated plates was carried out with hexane/ethyl acetate (19/1, v/v), twice in the first dimension, followed by petroleum ether/diethyl ether (17/3, v/v) three times in the second dimension.

### Drug Susceptibility Testing

Susceptibility of *M. tuberculosis* mc^2^7000 and clinical isolates to the various TAC analogues was determined on Middlebrook 7H10 solid medium containing OADC enrichment with increasing drug concentrations as reported earlier [17]. Serial 10-fold dilutions of each actively growing culture were plated and incubated at 37°C for two weeks. The minimal inhibitory concentration (MIC) was defined as the minimum concentration required to inhibit 99% of the growth.

### Reagents and Apparatus

Melting point determination was performed using a Stuart Scientific Melting Point SMP1 apparatus with degrees Celsius as the unit. Infra-red spectra were run on Jasco FT-IR-4200 ATR (Attenuated Total Reflection Mode) and Mattson Genesis Series FT-IR spectrometers with samples compressed with KBr into disks. Wavenumbers, (ν_max_) are expressed as cm^–1^. Proton nuclear magnetic resonance (^1^H NMR) and carbon (^13^C NMR) spectra were run on a JEOL Lambda delta 400 (400 MHz) spectrometer. Chemical shifts are stated in parts per million (ppm) and multiplicity indicated as singlet (s), doublet (d), triplet (t), quartet (q), and multiplet (m). Coupling constants (J) are quoted in Hertz (Hz) and deuterated solvents specified for each of the compounds. Elemental analysis was performed on Perkin Elmer 2400 Analyzer. Standard methods were used to determine C, H, and N simultaneously and halogens separately. Unless otherwise stated, all reagents and solvents were obtained from commercial sources. Solvents were dried according to standard procedures when deemed necessary.

### Chemical Synthesis of TAC Analogues

#### Synthesis of benzylidienehydrazinecarbothiamide (1) as a general procedure for the preparation of TAC analogues

Thiosemicarbazide, or derivative thereof, (0.91 g, 10 mmol, 1.0 eq) and glacial acetic acid (0.5 ml) were added to a stirring solution of benzaldehyde (1.1 g, 10 mmol, 1.0 eq) dissolved in absolute ethanol (10 ml). The mixture was refluxed for 3 hours before cooling and standing for 18 hours to afford a crude white powder. The solid was recrystalised using ethanol (95%), filtered and washed with cold ethanol to give **1** as a pure white solid (1.20 g 67.4%). m.p 163–164°C; ^1^H NMR (400 MHz, DMSO-*d*_6_) δ 9.70 (1H, s), 7.86 (1H, s), 7.63 (2H, d, J = 8.3 Hz), 7.41 (3H, d, J = 8.3 Hz), 6.44 (1H, s); ^13^C NMR (400 MHz, DMSO-*d*_6_) δ 182.9 (C = S), 145.1 (CH = N), 131.5 (Ar), 128.9 (Ar), 127 (Ar), 117.4 (Ar); υ_max_ (cm^–1^): 3417.2, 3399.9, 3229.2 (NH), 1598 C = N bend, 1282 C = S stretch; CHN analysis Found: C, 53.65; H, 4.59; N, 23.36; S, 17.62. Required for C_8_H_9_N_3_O_1_S: C, 53.63; H, 5.03; N, 23.46; S, 17.88.

#### Synthesis of 4-methylbenzylidenehydrazinecarbothioamide (2)

The title compound was obtained as a white solid (1.6 g, 79.2%) using the general procedure.m.p 175–177°C; ^1^H NMR (400 MHz, DMSO-*d*_6_) δ 9.67 (2H, s), 7.85 (1H, s), 7.54 (2H, d, J = 8.4 Hz), 7.21 (2H, d, J = 8.4 Hz), 6.37 (1H, s), 2.38 (3H, s); ^13^C NMR (400 MHz, DMSO-*d*_6_) δ 178.3 (C = S), 142.9 (C = N); 132.0 (Ar); 129.8 (Ar); 127.8 (Ar), 21.60 (CH); υ_max_ (cm^–1^): 3380, 3236 (NH), 1595 (C = N), 1177 (C = S); CHN analysis Found: C, 55.96; H, 5.43; N, 21.47; S, 16.12 Required for C_9_H_11_N_3_S: C, 55.96; H, 5.70; N, 21.76; S, 16.58.

#### Synthesis of 4-methylbenzylidene-N-methyl-hydrazinecarbothioamide (3)

The title compound was obtained as a white solid (1.1 g, 71.5%) using the general procedure. m.p 181–182°C; ^1^H NMR (400 MHz, DMSO-*d*_6_) δ 10.45 (1H,s), 8.42 (1H,s), 7.78 (2H, d, J = 8.2 Hz), 7.41 (2H, d, J = 8.2 Hz), 2.92 (3H, s), 2.31 (3H, s), 2.03 (2H, m); ^13^C NMR (400 MHz, DMSO-*d*_6_) δ 178.4 (C = S), 147.0 (C = N), 140.9 (Ar), 132.6 (AR), 128.6 (Ar), 127, 3 (Ar), 31.5 (CH_3_), 21.7 (CH_3_); υ_max_ (cm^–1^): 3422.7, 3274.2 (NH); 1525.1 (C = N); 1211.9 (C = S); CHN analysis Found: C, 57.89; H, 6.43; N, 20.45; S, 15.98. Required for C_10_H_13_N_3_S_,_ C, 57.94; H, 6.32; N, 20.27; S, 15.43.

#### Synthesis of 4-methylbenzylidene-N-phenyl-hydrazinecarbothioamide (4)

The title compound was obtained as a white solid (1.2 g, 68.9%) using the general procedure. m.p 192–193°C; ^1^H NMR (400 MHz, DMSO-*d*_6_) δ 10.75 (1H,s), 8.48 (1H,s), 7.72 (4H, m), 7.21 (4H, m), 6.82 (1H, m), 2.34 (3H, s), 2.03 (2H, m); ^13^C NMR (400 MHz, DMSO-*d*_6_) δ 179.2 (C = S), 147.0 (C = N), 141.5 (Ar), 140.6 (Ar), 129.5 (Ar), 128.4 (Ar), 126.6 (Ar), 125.7 (Ar), 122, 6 (Ar), 21.5 (CH_3_); υ_max_ (cm^–1^): 3437.6, 3239.2 (NH); 1578.9 (C = N); 1289.9 (C = S); CHN analysis Found: C, 67.01; H, 5.68; N, 15.82; S, 11.87. Required for C_15_H_15_N_3_S_,_ C, 66.88; H, 5.61; N, 15.60; S, 11.90.

#### Synthesis of 4-Ethylbenzylidenehydrazinecarbothioamide (5)

The title compound was obtained as a white solid (0.94 g, 44.2%) using the general procedure. m.p 133–134°C; ^1^H NMR (400 MHz, DMSO-*d*_6_) δ 10.01 (1H, s), 7.92 (s, 1H), 7.56 (2H, d, J = 8.2 Hz), 7.24 (2H, d, J = 8.2 Hz), 6.54 (2H, s), 2.66 (2H, d, J = 7.6 Hz), 1.26 (3H, t, J = 7.6 Hz); ^13^C NMR (400 MHz, DMSO-*d*_6_) δ 178.37 (C = S), 146.45 (CH = N), 142.90 (Ar), 132.32 (Ar), 128.67 (Ar), 127.94 (Ar), 28.67 (alk), 15.98 (alk); υ_max_ (cm^–1^): 3402, 3248, 3154.5 (NH); 1594 cm^–1^ C = N; 1534 cm^–1^ C = S; CHN analysis: Found: C, 57.56; H, 5.93; N, 20.09; S, 15.33. Required for C_10_H_13_N_3_S_1_: C, 57.97; H, 6.28; N, 20.29; S, 15.46.

#### Synthesis of N-methyl-2-(4-ethylbenzylidene)hydrazinecarbothioamide (6)

The title compound was obtained as a white solid (1.3 g, 74.6%) using the general procedure. m.p 183–184°C; ^1^H NMR (400 MHz, DMSO-*d*_6_) δ 10.12 (1H,s), 8.42 (1H,s), 7.82 (2H, d, J = 8.4 Hz), 7.36 (2H, d, J = 8.4 Hz), 2.92 (3H, s), 2.63 (2H, q, J = 7.2 Hz), 2.10 (2H, s), 1.32 (3H, t, J = 7.2 Hz); ^13^C NMR (400 MHz, DMSO-*d*_6_) δ 178.4 (C = S), 147.0 (C = N), 146.8 (Ar), 139.5 (Ar), 129.3 (Ar), 128.4 (Ar), 31.4 (alk), 28.4 (alk), 14.8 (alk); υ_max_ (cm^–1^): 3359.4, 3430.7 (NH); 1567.3 (C = N); 1235.7 (C = S); CHN analysis Found: C, 60.02; H, 6.75; N, 18.68; S 13.97. Required for C_11_H_15_N_3_S_,_ C, 59.69; H, 6.83; N, 18.99; S, 14.49.

#### Synthesis of 4-ethylbenzylidene-N-phenyl-hydrazinecarbothioamide (7)

The title compound was obtained as a white solid (1.5 g, 56.7%) using the general procedure. m.p 189–191°C; ^1^H NMR (400 MHz, DMSO-d_6_) δ 10.12 (1H,s), 8.42 (1H,s), 7.76 (4H. m), 7.32 (2H, d, J = 8.4 Hz), 7.22 (2H, t, J = 8.4 Hz), 6.92 (1H, m), 4.23 (1H, s), 2.64 (2H, q, J = 6.8 Hz), 2.13 (1H,s), 1.32 (3H, t, J = 6.8 Hz); ^13^C NMR (400 MHz, DMSO-d_6_) δ 178.4 (C = S), 147.0 (C = N), 146.8 (Ar), 139.6 (Ar), 139.5 (Ar), 130.9 (Ar), 129.4 (Ar), 129.6 (Ar), 128.9 (Ar), 127.8 (Ar), 28.9 (alk), 14.7 (alk); υ_max_ (cm^–1^): 3475.4, 3260.1 (NH); 1525.9 (C = N); 1269.0.2 (C = S); CHN analysis Found: C, 67.92; H, 6.12; N, 14.74; S 11.03. Required for C_16_H_17_N_3_S_,_ C, 67.81; H, 6.05; N, 14.83; S, 11.31.

#### Synthesis of 4-propylbenzylidenehydrazinecarbothioamide (8)

The title compound was obtained as a white solid (0.82 g, 37.1%) using the general procedure. m.p 148–149°C; ^1^H NMR (400 MHz, DMSO-*d*_6_) δ 11.40 (1H, s), 8.17 (1H, s), 7.95 (1H, s), 7.70 (2H, d, J = 8.3 Hz), 7.22 (2H, d, J = 8.3 Hz), 2.13 (2H, t, J = 7.4 Hz), 1.59 (2H, m), 0.89 (3H, t, J = 7.6 Hz); ^13^C NMR (400 MHz, DMSO-*d*_6_) δ 178.44 (C = S), 144.92 (Ar), 142.97 (CH = N), 132.41 (Ar), 129.32 (Ar), 127.93, 31.33 (alk), 24.58 (alk), 14.27 (alk); υ_max_ (cm^–1^): 3402, 3215, 3156 (NH); 1594 (C = N); 1291 (C = S); CHN analysis: Found: C, 59.68; H, 6.39; N, 19.00; S, 14.48; Required for C_11_H_15_N_3_S_1_: C, 59.73; H, 6.79; N, 19.00; S, 14.48.

#### Synthesis of 4-isopropylbenzylidenehydrazinecarbothioamide (9)

The title compound was obtained as a white solid (0.68 g, 30.8%) using the general procedure.m.p 151–153°C; ^1^H NMR (400 MHz, DMSO-*d*_6_) δ 9.37 (1H, s, NH_2_), 7.89 (1H,s), 7.57 (2H, d, J = 8.3 Hz), 7.25 (2H, d, J-8.3 Hz), 6.46 (2H, s), 2.94 (1H, m), 1.25 (6H, s); ^13^C NMR (400 MHz, DMSO-*d*_6_) δ 178.4 (C = S), 151.0 (Ar), 142.9 (Ar), 132.0 (Ar), 128.9 (Ar), 127.2 (Ar), 34.3 (CH_3_)_2_), 23.84 (CH); υ_max_ (cm^–1^): 3409, 3276, 3152 (NH); 1599 (C = N); 1281.9 (C = S); CHN analysis Found: Found: C, 59.54; H, 6.22; N, 19.01; S, 14.38 Required for C_11_H_15_N_3_S: C, 59.73; H, 6.79; N, 19.00; S, 14.48.

#### Synthesis of 2-methoxybenzylidenehydrazinecarbothioamide (10)

The title compound was obtained as a white solid (1.6 g, 85.3%) using the general procedure. m.p 212–213°C; ^1^H NMR (400 MHz, DMSO-*d*_6_) δ 8.32 (1H, s), 8.51 (2H, s), 7.65 (1H, d, J = 8.1 Hz), 7.55 (1H, m), 7.23 (1H, d, J = 8.4 Hz), 7.12 (1 H, t, J = 8.1 Hz), 3.88 (3H, s), 2.8 (1H,s); ^13^C NMR (400 MHz, DMSO-*d*_6_) δ 179.1 (C = S), 160.1 (Ar), 145.2 (C = N), 134, 2 (Ar), 132.1 (Ar), 130.5 (Ar), 116.7 (Ar), 110.5 (Ar), 55.3 (OCH_3_); υ_max_ (cm^–1^): 3432.0, 3273.9, 3153.2 (NH); 1583 cm^–1^ (C = N); 1274 cm^–1^ (C = S); CHN analysis Found: C51.44,; H, 4.53; N, 19.97; S, 15.88.Required for C_9_H_11_N_3_OS: C, 51.65; H, 5.30; N, 20.08; S, 15.38.

#### Synthesis of 3-methoxybenzylidenehydrazinecarbothioamide (11)

The title compound was obtained as a white solid (1.2 g, 75.0%) using the general procedure. m.p 194–195°C; ^1^H NMR (400 MHz, DMSO-*d*_6_) δ 11.45 (1H,s); 8.25 (1H s) 8.08 (1H, s) 8.00 (1H, d, J = 8.4 Hz), 7.44 (1H, d, J-8.4 Hz); 7.27 (1H, t, J = 8.3 Hz); 6.95 (1H, d, J = 8.3 Hz); 3.79 (3H, s), 2.3 (1H, s); ^13^C NMR (400 MHz, DMSO-*d*_6_) δ 179.3 (C = S), 162.1 (Ar), 146.2 (C = N), 131.2 (Ar), 129.3 (Ar), 128.2(Ar), 112.7 (Ar), 113.2 (Ar), 54.9 (OCH_3_); υ_max_ (cm^–1^): 3433.1, 3273.5, 3148.2 (NH); 1587.5 cm^–1^ (C = N); 1269.2 cm^–1^ (C = S); CHN analysis Found: C51.44,; H, 4.97; N, 20.15; S, 15.61.Required for C_9_H_11_N_3_OS: C, 51.65; H, 5.30; N, 20.08; S, 15.38.

#### Synthesis of 4-methoxybenzylidenehydrazinecarbothioamide (12)

The title compound was obtained as a white solid (1.7 g, 83.0%) using the general procedure. m.p 167–168°C; ^1^H NMR (400MHz, DMSO-*d*_6_) δ 11.30 (1H, s), 8.12 (1H, s), 7.99 (1H, s), 7.92 (1H, s), 7.57 (2H, d, J = 8.2 Hz), 6.96 (2H, d, J = 8.2 Hz), 3.79 (3H, s); ^13^C NMR (400 MHz, DMSO-*d*_6_) δ 179.3 (C = S), 160 (Ar); 142.5 (C = N); 114.7 (Ar); 126 (Ar); 129.47 (Ar), 56.1 (OCH_3_); υ_max_ (cm^–1^): 3397, 3248.2,(NH); 1596 (C = N), 1232 (C = S); CHN analysis Found C, 50.63 H, 5.30, N, 20.14 S, 15.25; Required for C_9_H_11_N_3_0_1_S_1_ (Required for C 51.97; H, 5.26; N, 20.09; S, 15.31.

#### Synthesis of 4-methoxybenzylidene-N-phenylhydrazinecarbothioamide (13)

The title compound was obtained as a white solid (1.9 g, 67.7%) using the general procedure. m.p 174–175°C; ^1^H NMR (400 MHz, DMSO-*d*_6_) δ 8.16 (1H, s), 7.76 (2H, d, J = 8.6 Hz), 7.59 (2H, d, J = 8.3 Hz), 7.13 (2H, t, J = 8.3 Hz), 7.02 (2H, d, J = 8.6 Hz), 6.74 (1H, s), 4.9 (1H, s), 3.76 (3H, s), 1.8 (1H, s); ^13^C NMR (400 MHz, DMSO-*d*_6_) δ 180.1 (C = S), 162.9 (Ar), 147.3 (C = N), 139.8 (Ar), 130.4 (Ar), 128.9 (Ar), 128.0 (Ar), 125.6 (Ar), 125.1 (Ar), 113.8 (Ar), 57.3 (OCH_3_); υ_max_ (cm^–1^): 3352.2, 3141.5 (NH) (NH), 1606 (C = N); 1242(C = S); CHN analysis Found: C, 63.15; H, 5.41; N, 14.82; S 11.65, (Required for C_15_H_1_ N_3_S), C, 63.13; H, 5.30; N, 14.73; S, 1.24).

#### Synthesis of 4-methoxybenzylidene-N-methylhydrazinecarbothioamide (14)

The title compound was obtained as a white solid (1.86 g, 80.1%) using the general procedure.m.p 202–203°C; ^1^H NMR (400 MHz, DMSO-*d*_6_) δ 11.38 (1H, s), 8.44 (1H, s), 7.99 (1H, s), 7.75 (2H, d, J-8.4Hz), 6.99 (2H, d, J = 8.4Hz), 3.01(3H, s), 3.80 (3H, s); ^13^C NMR (400 MHz, DMSO-*d*_6_) δ 178.5 (C = S), 160.4 (Ar), 141.0 (C = N), 129.4 (Ar), 114.2 (Ar), 55.87 (OCH_3_), 15.28 (CH_3_-NH); υ_max_ (cm^–1^): 3317.4, 3154 (NH),; 1599 (C = N); 1251(C = S); CHN analysis Found: C53.87; H, 5.99; N, 18.89; S 14.68,. (Required for C_10_H_13_N_3_0S, C, 53.81; H, 5.81, N, 18.83, S, 14.35.

#### Synthesis of 4-Ethoxybenzylthiosemicarbamide (15)

The title compound was obtained as a white solid (1.4 g, 33.9%) using the general procedure. m.p 155–157°C; ^1^H NMR (400 MHz, DMSO-*d*_6_) δ 11.25 (1H, s), 8.12 (2H, s,), 8.0 (2H, s,); 7.73 (2H, d, J = 8.3 Hz), 6.95 (2H, d, J = 8.3 Hz), 4.05(2H, q, J = 7.4Hz), 1.33(3H, t, J = 7.4Hz.; ^13^C NMR (400MHz, DMSO-*d*_6_) δ 178.14 (C = S), 160.53 (Ar); 142.80 (C = N), 129.49 (Ar), 127.15 (Ar), 115.11 (Ar); 63.78 (Alk), 15.15 (Alk); υ_max_ (cm^–1^): 3419.7, 3244.2 (NH); 1170.1 (C = S); 1595 (C = N); CHN analysis Found: C, 53.82; H, 5.75; N, 18.91; S, 14.59. Required for C_10_H_13_N_3_OS_,_ C, 53.79; H, 5.87; N, 18.82; S, 14.36).

#### Synthesis of 4-ethoxybenzylidenehydrazinecarbothioamide (16)

The title compound was obtained as a white solid (1.2 g, 45.2%) using the general procedure. m.p 156–157°C; ^1^H NMR (400MHz, DMSO-*d*_6_) δ 11.40 (2H, s) 8.11 (1H, s), 7.98 (1H, s), 7.71 (2H, d, J = 8.3 Hz); 6.9 (2H, d, J = 8.3Hz); 4.1 (2H, t, 7.4 Hz); 2.01 (2H, p, J = 7.5 Hz), 1.32 (3H, t, 7.4Hz); ^13^C NMR (400 MHz, DMSO-*d*_6_) δ; 179.2 (C = S), 161.3 (Ar), 147.4 (C = N), 130.3 (Ar), 125.8 (Ar), 118.3 (Ar), 71.2 (alk), 24, 2 (alk), 10.1 (alk); υ_max_ (cm^–1^): 3434.0, 32765.9, 3153.2 (NH); 1594 cm^−1^ (C = N); 1287 cm^−1^ (C = S); CHN analysis Found: C, 53.73; H, 5.69; N, 18.70; S, 14.30. Required for C_11_H_15_N_3_OS: C, 53.79; H, 5.87; N, 18.82; S, 14.36).

#### Synthesis of 4-Butoxybenzylthiosemicarbamide (17)

The title compound was obtained as a white solid (3.1 g, 70.7%) using the general procedure. m.p 170–176°C; ^1^H NMR (400MHz, DMSO-*d*_6_) δ 11.26 (1H, s); 8.12, 7.92 (1H, s); 8.0(2H,s); 7.71 (2H, d, J = 8.4Hz); 6.95 (2H, d, J = 8.4Hz); 3.98(2H, t, J = 7.6Hz); 1.68 (2 H, q, J = 7.6Hz); 1.43 (2H, sext, J = 7.6Hz); 0.92 (3H, t, J = 7.4Hz); ^13^C NMR (400MHz, DMSO-*d*_6_) δ 178.1 (C = S), 142.8 (C = N), 160.71 (Ar), 129, 54 (Ar), 127.15 (Ar), 115.14 (Ar), 67.84 (alk), 31.25 (alk), 19.29 (alk); υ_max_ (cm^−1^): 3425.7, 3221.2 (NH); 1565.4 (C = N); 1241.1 (C = S); CHN analysis Found: C, 57.29; H, 6.90; N, 16.89; S, 13.01. Required for C_12_H_17_N_3_OS_,_ C, 57.34; H, 6.82; N, 16.72; S, 12.76).

#### Synthesis of 4-phenoxybenzylidenehydrazinecarbothioamide (18)

The title compound was obtained as a white solid (1.85 g, 68.3%) using the general procedure. m.p 145–146°C; ^1^H NMR (400MHz, DMSO-*d*_6_) δ 10.01 (1H, s), 8.17 (3H, m); 7.82 (2H, d, J = 8.4Hz); 7.42 (2H, m); 7.08 (5H, m); ^13^C NMR (400MHz, DMSO-*d*_6_) δ and 159.1, 158.2 (Ar), 141 (C = N), 130.8 (Ar), 129.8 (Ar), 179 (C = S), 124.6 (ArC = N), 118.81 (Ar), 119.85 (Ar); υ_max_ (cm^−1^): 3387, 3245, 3143 (NH); 1587 (C = N), 1238 (C = S); CHN analysis Found: Found: C, 65.78; H, 4.75; N, 15.98; Required for C_14_H_13_N_3_S: C, 65.85; H, 5.13; N, 16.46, S, 12.56.

#### Synthesis of 2-Flurobenzylidenehydrazinecarbothioamide (19)

The title compound was obtained as a white solid (0.46 g, 24%) using the general procedure.m.p 184–186°C; ^1^H NMR (400MHz, DMSO-*d*_6_) δ 11.58 (1H, s); 8.27 (2H, m), 8.10(1H, s), 7.44 (2H, m, CH), 7.24 (2H, m); ^13^C NMR (400MHz, DMSO-*d*_6_) δ; 178.2 (C = S), 160.3 (Ar), 143.5 (C = N), 133.5 (Ar), 130.2 (Ar), 125.6 (Ar), 120.5 (Ar), 120.1 (Ar), 115.5 (Ar); υ_max_ (cm^−1^): 3433.4, 3254.1 (NH), 1592.5 (C = N), 1175 (C = S); CHN analysis Found: C, 48.37; H, 3.54; N, 20.99; S, 16.59; (Required for C_9_H_9_N_3_SF: C, 48.69; H, 4.09; N, 21.31; S, 16.27).

#### Synthesis of 3-Flurobenzylidenehydrazinecarbothioamide (20)

The title compound was obtained as a white solid (0.5 g, 26%) using the general procedure. m.p 188–189°C; ^1^H NMR (400MHz, DMSO-*d*_6_) δ 11.53 (1H, s), 8.28 (1H, s), 8.18 (1H, s), 8.02 (1H, s), 7.83 (1H, d, J = 8.4Hz), 7.51 (1H, d, J = 8.4Hz), 7.44 (1H, m), 7.21 (1H, t, J = 8.5 Hz); ^13^C NMR (400MHz, DMSO-*d*_6_) δ; 178.1 (C = S), 154.3 (Ar), 143.1 (C = N), 132.9 (Ar), 131.0 (Ar), 127.7 (Ar), 126.2 (Ar), 122.3 (Ar), 113.5 (Ar); υ_max_ (cm^−1^): 34329.4, 3253.9, (NH), 1590.5 (C = N), 1172.1 (C = S); CHN analysis Found: C, 48.36; H, 3.81; N, 21.22; S, 16.65; (Required for C_9_H_9_N_3_SF: C, 48.69; H, 4.09; N, 21.31; S, 16.27).

#### Synthesis of 4-flurobenzylidenehydrazinecarbothioamide (21)

The title compound was obtained as a white solid (0.8 g, 43.2%) using the general procedure. m.p 189–191°C; ^1^H NMR (400MHz, DMSO-*d*_6_) δ 11.55 (1H, s) 8.22 (1H, s), 8.04 (2H, s), 7.88 (2H, d, J = 8.4Hz), 7.24 (2H, d, J = 8.4Hz), ^13^C NMR (400MHz, DMSO-*d*_6_) δ; 178.1 (C = S), 154.3 (Ar), 143.1 (C = N), 132.9 (Ar), 131.0 (Ar), 127.7 (Ar), 126.2 (Ar), 122.3 (Ar), 113.5 (Ar); υ_max_ (cm^−1^): 3432.94, 3252.1, (NH), 1592.1 (C = N), 1170.9 (C = S); CHN analysis Found: C, 48.51; H, 3.61; N, 21.08; S, 16.46; (Required for C_9_H_9_N_3_SF: C, 48.69; H, 4.09; N, 21.31; S, 16.27).

#### Synthesis of 4-fluorobenzylidene-N-methylhydrazinecarbothioamide (22)

The title compound was obtained as a white solid (0.7 g, 65.7%) using the general procedure.m.p 194–195°C; ^1^H NMR (400 MHz, DMSO-*d*_6_) δ 10.01 (1H,s), 8.43 (1H,s), 7.86 (2H, d, J = 8.4Hz), 7.38 (2H, d, J = 8.4 Hz), 2.81 (3H, t, J = 7.2Hz); (2.01 (2H, s); ^13^C NMR (400MHz, DMSO-*d*_6_) δ 178.4 (C = S), 163.6 (Ar), 147.0 (C = N), 143.4 (Ar), 138.2 (Ar), 128.3 (Ar), 31.4 (CH_3_); υ_max_ (cm^−1^): 3325.3, 3466.4 (NH); 1527.1 (C = N); 1267.2 (C = S); CHN analysis Found: C, 51.23; H, 4.76; N, 19.21; S 14.92. Required for C_9_H_10_FN_3_S_,_ C, 51.17; H, 4.77; N, 19.89; S, 15.18.

#### 4-fluorobenzylidene-N-phenylhydrazinecarbothioamide (23)

The title compound was obtained as a white solid (0.7 g, 66.6%) using the general procedure.m.p 198–200°C; ^1^H NMR (400MHz, DMSO-*d*_6_) δ 10.21 (1H,s), 8.43 (1H,s), 7.82 (2H, d, J = 8.4Hz), 7.74 (2H, d, J = 8.4 Hz), 7.36 (2H, m), 7.21 (2H, m), 6.83 (1H, t, J = 8.5 Hz), (1.97 (2H, s); ^13^C NMR (400MHz, DMSO-*d*_6_) δ 178.4 (C = S), 165.7 (Ar), 147.0 (C = N), 138.5 (Ar), 130.6 (Ar), 129.6 (Ar), 129.2 (Ar), 129.0 (Ar), 126.6 (Ar), 117.6 (Ar); υ_max_ (cm^−1^): 3329.1, 3423.6 (NH); 1565.9 (C = N); 1237.3 (C = S); CHN analysis Found: C, 61.58; H, 4.56; N, 15.65; S 11.89. Required for C_14_H_12_F N_3_S_,_ C, 61.52; H, 4.43; N, 15.37; S, 11.73.

#### Synthesis of 2-chlorobenzylidenehydrazinecarbothioamide (24)

The title compound was obtained as a white solid (1.2 g, 70.1%) using the general procedure. m.p 210–211°C; ^1^H NMR (400MHz, DMSO-*d*_6_) δ 11.5 (1H, s), 8.47 (1H, s), 8.30 (2H, s)_,_ 8.13 (1H, m), 7.48 (1H, m), 7.46 (1H, m), 7.36 (1H, m); ^13^C NMR (400MHz, DMSO-*d*_6_) δ 178.5 (C = S), 139.4 (C = N), 135.6 (Ar), 134.7 (Ar), 132.5 (Ar), 130.1 (Ar), 127.5 (Ar), 126.9 (Ar), 125.7 (Ar); υ_max_ (cm^−1^): 3414.3, 3153.4 (NH); 3153.44, 3248.85 (NH); 1610.59 (C = N); 1278.34 (C = S); CHN analysis Found: C, 44.79; H, 3.73; N, 19.52; S, 15.59. Required for C_8_H_9_N_3_ClS_,_ C, 44.96; H, 3.75; N, 19.67; S, 14.99).

#### Synthesis of 3-chlorobenzylidenehydrazinecarbothioamide (25)

The title compound was obtained as a white solid (1.1 g, 44.1%) using the general procedure. m.p 200–202°C; ^1^H NMR (400MHz, DMSO-*d*_6_) δ 11.5 (1H, s), 8.47 (1H, s), 8.30 (2H, s)_,_ 8.13 (1H, m), 7.48 (1H. m), 7.46 (1H, m), 7.36 (1H, m); ^13^C NMR (400MHz, DMSO-*d*_6_) δ 178.5 (C = S), 139.4 (C = N), 135.6 (Ar), 134.7 (Ar), 132.5 (Ar), 130.1 (Ar), 127.5 (Ar), 126.9 (Ar), 125.7 (Ar); υ_max_ (cm^−1^): 3390.3, 3152.9 (NH); 1602.6 (C = N); 1165.3 (C = S); CHN analysis Found: C, 44.88; H, 3.61; N, 19.51; S, 14.92. Required for C_8_H_9_N_3_ClS_,_ C, 44.96; H, 3.75; N, 19.67; S, 14.99).

#### Synthesis of 4-chlorobenzylidenehydrazinecarbothioamide (26)

The title compound was obtained as a white solid (1.6 g, 74%) using the general procedure.m.p 202–203°C; ^1^H NMR (400MHz, DMSO-*d*_6_) δ 11.58 (1H, s), 8.24 (1H, s), 8.08 (1H, s,), 8.02 (1H,s), 7.84 (2H, d, J = 8.3Hz), 7.45 (2H, d, J = 8.3Hz); ^13^C NMR (400MHz, DMSO-*d*_6_) δ 178.6 (C = S), 141.4 (C = N), 134.7 (Ar), 133.7 (Ar), 129.5 (Ar), 129.2 (Ar); υ_max_ (cm^−1^): 3436.0, 3277.9, 3163.2 (NH); 1598 cm^−1^ (C = N); 1282 cm^−1^ (C = S); CHN analysis Found: C, 44.92; H, 3.86; N, 20.06; S, 15.21. Required for C_8_H_8_N_3_ClS: C, 44.97; H, 3.77; N, 19.66; S, 15.01.

#### Synthesis of 4-chlorobenzylidene-N-methylhydrazinecarbothioamide (27)

The title compound was obtained as a white solid (2.1 g, 90.1%) using the general procedure. m.p 205–206°C; ^1^H NMR (400MHz, DMSO-*d*_6_) δ 11.49 (1H, s), 8.58 (1H, d, J = 8.6), 8.01 (1H, s), 7.83 (2H, d, J = 8.3), 7.47 (2H, J = 8.3), 3.01 (3H, d, J = 7.8); ^13^C NMR (400MHz, DMSO-*d*_6_) δ 179.8 (C = S), 134.7 (Ar); 140.8 (CH = N); 129.27 (Ar), 39.65 (CH_3_); υ_max_ (cm^−1^): 3345.4, 3152NH (NH)s; 1594 (C = N), 1261 (C = S); CHN analysis Found: C, 47.09; H, 4.35; N, 18.46; S, 13.99; C_10_H_12_Cl_1_N_3_S_1_; Required for C_12_H_11_N_3_S_1_: C, 47.47; H, 4.40; N, 18.24; S, 14.07.

#### Synthesis of 4-chlorobenzylidene-N-phenylhydrazinecarbothioamide (28)

The title compound was obtained as a white solid (2.5 g, 85.5%) using the general procedure.m.p 192–193°C; ^1^H NMR (400MHz, DMSO-*d*_6_) δ 11.89 (1H, s), 10.19 (1H, s,), 8.13 (1H, s), 7.97 (2H, J = 8.4Hz); 7.50 (4H, t, J-8.3 Hz); 7.40 (2H, t, J = 8.3Hz); 7.21 (1H, d, J = 8.4 Hz); ^13^C NMR (400MHz, DMSO-*d*_6_) δ 176.7 (C = S), 142.0 (C = N), 139.6 (Ar), 126.0 (Ar), 135.3 (Ar); 126.7 (Ar); υ_max_ (cm^−1^): 3308, 3132 (NH); 1592 (C = N), 1260 (C = S); CHN analysis Found: C, 58.03; H, 4.15; N, 14.51; Cl, 12.26; S, 11.05; C_10_H_12_Cl_1_N_3_S_1_; Required for C_12_H_11_N_3_S_1_: C, 57.51; H, 4.14; N, 14.44; Cl, 12.01; S, 10.56.

#### Synthesis of 2-bromobenzylidenehydrazinecarbothioamide (29)

The title compound was obtained as a white solid (0.53 g, 67.8%) using the general procedure. m.p 202–203°C; ^1^H NMR (400MHz, DMSO-*d*_6_) δ 11.7 (1H, s) 8.43 (2H, s); 8.29 (1H, t, J = 8.5Hz); 8.28 (2H, t, J = 8.4Hz); 8.12 (1H, s); 7.64 (1H, d, J-8.5Hz); ^13^C NMR (400MHz, DMSO-*d*_6_) δ 178.4 (C = S), 143.8 (C = N), 136.7 (Ar), 136.1 (Ar), 133.1 (Ar), 130.2 (Ar), 128.6 (Ar), 120.1 (Ar); υ_max_ (cm^−1^): 3435.9; 3285.1 (NH); 1599.6 (C = N); 1170.3 (C = S); CHN analysis Found: C, 37.17; H, 2.84; N, 16.13; S, 12.66. Required for BrC_8_H_8_N_3_: S: C, 37.1; H, 3.09; N, 16.2; S, 12.4.

#### Synthesis of 3-bromobenzylidenehydrazinecarbothioamide (30)

The title compound was obtained as a white solid (1.1 g, 72.3%) using the general procedure. m.p 212–213°C; ^1^H NMR (400MHz, DMSO-*d*_6_) δ 11.51 (1H, s); 8.29 (2H, s); 8.2 (1H, d, J = 8.4 Hz); 8.0 (1H, s); 7.69 (1H, d, J = 8.3Hz); 7.56 (1H, d, J = 8.3Hz); 7.35 (1H, t, J = 8.4Hz); ^13^C NMR (400MHz, DMSO-*d*_6_) δ 178.2 (C = S), 143.5 (C = N), 135.7 (Ar), 134.2 (Ar), 134.0 (Ar), 132.2 (Ar), 129.6 (Ar), 122.1 (Ar); υ_max_ (cm^−1^): 3434.2; 3282.4 (NH); 1598.9 (C = N); 1168.9 (C = S); CHN analysis Found: C, 37.18; H, 2.81; N, 16.13; S, 12.88. Required for BrC_8_H_8_N_3_: S: C, 37.1; H, 3.09; N, 16.2; S, 12.4.

#### Synthesis of 4-bromobenzylidenehydrazinecarbothioamide (31)

The title compound was obtained as a white solid (1.2 g, 69.5%) using the general procedure. m.p 225–226°C; ^1^H NMR (400MHz, DMSO-*d*_6_) δ 11.66 (1H, s) 8.43 (2H, s); 8.33 (2H, d, J = 8.4Hz); 8.28 (2H, d, J = 8.4Hz); 8.13 (1H, s); ^13^C NMR (400MHz, DMSO-*d*_6_) δ 178.0 (C = S), 143.0 (C = N), 135.7 (Ar), 133.6 (Ar), 133.3 (Ar), 130.5 (Ar), 129.9 (Ar), 120.4 (Ar); υ_max_ (cm^−1^): 3435.1; 3280.2 (NH); 1593.5 (C = N); 1170.4 (C = S); CHN analysis Found: C, 37.24; H, 2.85; N, 16.00; S, 13, 23. Required for BrC_8_H_8_N_3_: S: C, 37.1; H, 3.09; N, 16.2; S, 12.4.

## Results and Discussion

### Isolation of *M. tuberculosis* Spontaneous Mutants Resistant to TAC and SRI-224

A TAC analogue, SRI-224, was previously tested against *Mycobacterium avium* and found to be more effective than TAC *in vitro* and in mice [30], and has also been shown to be very active against *M. tuberculosis* [20]. To continue our studies on the antitubercular activity of TAC, we selected TAC-resistant *M. tuberculosis* mutants by plating actively-growing cultures on Middlebrook 7H10 media supplemented with OADC and either TAC or SRI-224 at concentrations that were 10 to 40 times higher than the minimum inhibitory concentration (MIC_99_). The ten mutants that were obtained, labeled MTTR2 through MTTR28, are listed in Table 2, which indicates the concentration of the drug on which these were selected and shows that most mutants exhibited comparably high levels of resistance to both TAC and SRI-224, with MIC values ≥20 µg/ml compared to the MIC of 0.25 µg/ml for the parental strain, as previously reported [23].

**Table 2:** Susceptibility and genetic mutations associated to TAC resistance in *M. tuberculosis* strains selected on high concentrations of TAC or SRI-224.

M. tuberculosis strains	Drug selection(µg/ml)	MIC TAC(µg/ml)	MIC SRI-224(µg/ml)	Ketomycolates	mmaA4 mutation(AA change)	ethA mutation(AA change)	hadA mutation(AA change)	hadB mutation(AA change)	hadC mutation(AA change)
**MTTR2**	TAC 2,5	>20	20	+	none	none	none	none	a367g (T123A)
**MTTR3**	TAC 2,5	>20	20	+	none	none	t181g (C61G)	none	none
**MTTR5**	TAC 2,5	>20	20	+	none	none	t181g (C61G)	none	none
**MTTR6**	TAC 2,5	20	20	+	none	none	t181a (C61S)	none	none
**MTTR11**	TAC 5	>20	20	+	none	none	t181g (C61G)	none	none
**MTTR13**	TAC 5	20	5	-	g302a (G101D)	none	none	none	none
**MTTR14**	TAC 5	>20	20	+	none	none	none	none	a470g (K157R)
**MTTR18**	SRI-224 2,5	20	10	+	none	none	none	none	g253t (V85F)
**MTTR19**	SRI-224 2,5	10–20	20	+	none	none	t181g (C61G)	none	none
**MTTR28**	SRI-224 10	>20	20	+	none	none	t181g (C61G)	none	none
**Parental**		0,25	0,1	+	none	none	none	none	none

### Mycolic Acid Profile in the *M. tuberculosis* TAC Resistant Mutants

**Figure 1:**
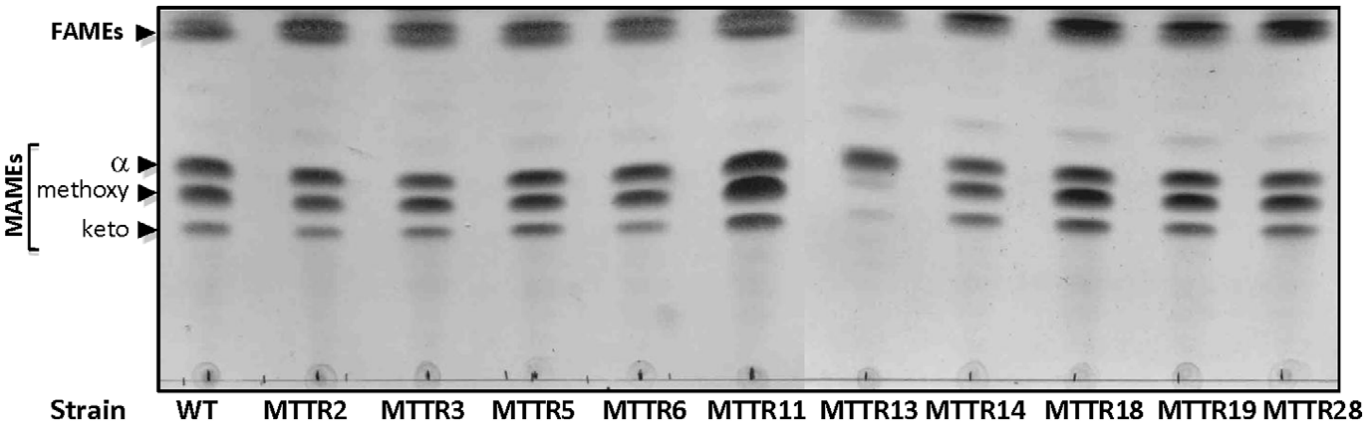
Mycolic acid profile of the parental *M. tuberculosis* strain and independent TAC-resistant derived mutants. FAMEs and MAMEs were extracted from exponentially growing cultures and resolved by single dimension TLC in hexane/ethyl acetate (19/1, v/v) prior to visualization using molybdophosphoric acid and charring. α, α-mycolic acids; methoxy, methoxy-mycolic acids; keto, keto-mycolic acids.

Several recent studies highlighted the possible link between TAC activity and the mycolic acid biosynthetic pathway. First, we originally demonstrated that TAC inhibits mycolic acid biosynthesis [17], although the profile was different from that generated with ETH treatment, a drug that is known to target the enoyl-ACP reductase of FAS-II. Second, we demonstrated that inhibition of cyclopropanation of mycolic acids is one of the effects of treatment with TAC or its analogue SRI-224, thus providing strong evidence linking the mode of action of TAC with mycolic acid metabolism [20]. Third, subsequent work identified an important set of TAC-resistant BCG and *M. tuberculosis* mutants that were lacking keto-mycolic acids due to mutations within the *mmaA4* gene [23]. Further, overexpression of *mmaA4* in BCG resulted in increased susceptibility to TAC. MmaA4 (also known as Hma) is an *S-*adenosylmethionine (SAM)-dependent methyltransferase required to produce the hydroxymycolate precursor of the oxygenated mycolic acid [15]. A recent study identified mutations in the β-hydroxyacyl ACP dehydratase complex of the FAS-II system capable of conferring resistance to TAC in *M. tuberculosis* and *M. kansasii* [24]. In addition, it was shown that exposure of *M. tuberculosis* to TAC was associated to an accumulation of 3-hydroxy fatty acids, the dehydratase substrates [25], thus providing an additional proof that activity of TAC is highly interconnected with mycolic acid biosynthesis [24]. This growing body of evidence connecting TAC activity and TAC-resistance mutations to mycolic acid metabolism prompted us to examine the mycolic acid profile of all 10 mutant strains (Fig. 1).

Fatty acid methyl esters (FAMEs) and mycolic acid methyl esters (MAMEs) were extracted from exponentially growing cultures, resolved by thin layer chromatography (TLC) and visualized following charring with molybdophosphoric acid. As shown in Fig. 1, all mutants exhibited a standard profile compared to the parental strain with the notable exception of MTTR13, which produces almost exclusively α-mycolic acids with very limited amounts of methoxy- and keto-mycolic acids. Because MmaA4 is required for the synthesis of oxygenated mycolic acids [15] and TAC-resistant strains lacking oxygenated mycolic acids have been identified previously, it seemed likely that MTTR13 had a mutation in *mmaA4*. However, as this was the only one of the ten TAC-resistant strains with this mycolic acid pattern, it was clear that other mechanisms of TAC resistance must be operating.

### Genotype of the TAC-associated Resistance Markers

To characterize these additional mutations conferring TAC resistance in our strains, we sequenced the genes encoding all proteins known to be associated with TAC resistance, namely *ethA, mmaA4* and *hadABC.* As shown in Table 2, no mutations were found in *ethA*, indicating that resistance was not mediated by a defect in the TAC activation process. As expected, a mutation in *mmaA4* was found only in mutant MTTR13, consistent with the lack of oxygenated mycolates in this strain (Fig. 1). Interestingly, the Gly101Asp mutation in MmaA4 had not been identified in BCG mutants resistant to TAC [23], but was found in an *M. tuberculosis* strain resistant to TAC [24].

Among the remaining nine strains that were not mutated in *mmaA4,* six (MTTR3, MTTR5, MTTR6, MTTR11, MTTR19, MTTR28) had mutations in *hadA*, and the remaining three (MTTR2, MTTR14, and MTTR18) harbored mutations in *hadC*. All of the *hadA* mutants were missense mutations at Cys61, replacing it either with a Gly or a Ser. The Cys61Ser substitution was described previously in a TAC-resistant mutant of *M. tuberculosis* and also a TAC-resistant *M. kansasii* strain [24]. In contrast, the missense mutations in *hadC* were more varied, with substitutions occurring in Val85, Lys157 and Thr123. Only the mutations in Val85 and Ala151 were reported previously in *M. kansasii* and *M. tuberculosis,* respectively, so our study extends the sites of mutations in HadC associated with TAC resistance. Mapping these mutations on the three-dimensional structures of HadA or HadC would greatly help to understand the impact of these mutations on dehydratase activity or the interactions of these proteins with other FAS-II components, but adequate structures of these proteins are not yet available. MTTR3, MTTR13 and MTTR18 grew similarly to the wild-type strain in broth medium, indicating that mutations in *hadA*, *hadC* or *mmaA4* do not confer a growth defect (data not shown). As in the study by Belardinelli and Morbidoni [24], we failed to identify missense mutations in *hadB*. Taken together, these results support the conclusion that resistance to TAC in *M. tuberculosis* can be attributed to missense mutations in either *mmaA4*, *hadA* or *hadC* and, at least in our mutant strains, the C61G mutation in HadA is the most common amino acid change associated with TAC resistance.

**Figure 2:**
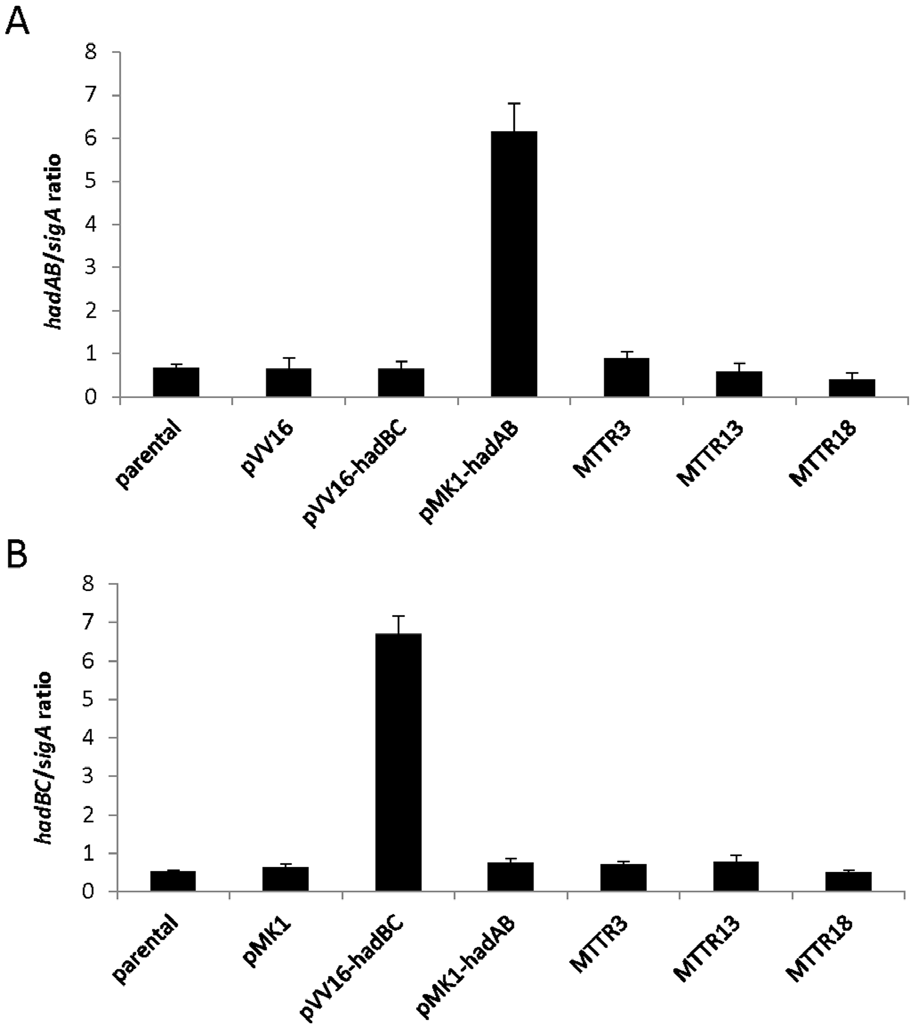
*HadAB* and *hadBC* expression levels in TAC-resistant *M. tuberculosis* mutants. The parental *M. tuberculosis* strain as well as MTTR3, MTTR13 or MTTR18 were grown to mid-log phase. Total RNA was isolated from three independent replicates and the levels of *hadAB* **(A)** and *hadBC* **(B)** transcripts relative to those of the s*igA* gene were measured by quantitative reverse transcription-PCR. Recombinant strains carrying the pVV16-*hadAB* or pVV16-*hadBC* constructs were included as positive controls of HadAB and HadBC overexpression, respectively.

Because drug resistance can be generated by increased levels of the specific target, we explored whether resistance to TAC in the MTTR mutants is associated with the overexpression of HadA and/or HadC. Quantitative real-time PCR was performed to determine the expression levels of the *hadA* (Fig. 2A) and *hadC* (Fig. 2B) transcripts in representative members of the different mutant classes: MTTR3 (containing the C61G substitution in HadA), MTTR13 (containing the G101D substitution in MmaA4) and MTTR18 (containing the V85F substitution in HadC). The results clearly demonstrate that in all three mutants the expression of *hadAB* and *hadBC* is comparable to that of the parental strain (Fig. 2), showing that point mutations in HadA or HadC do not alter their levels expression. In addition, these results exclude the presence of additional mutations within the *hadABC* operon driving overexpression of the target genes, as has been described for the C15T *inhA* promoter mutation that confers INH resistance by increasing the transcription of *inhA* [5].

### Synthesis and Activity of TAC-related Analogues against *M. tuberculosis*

**Figure 3:**
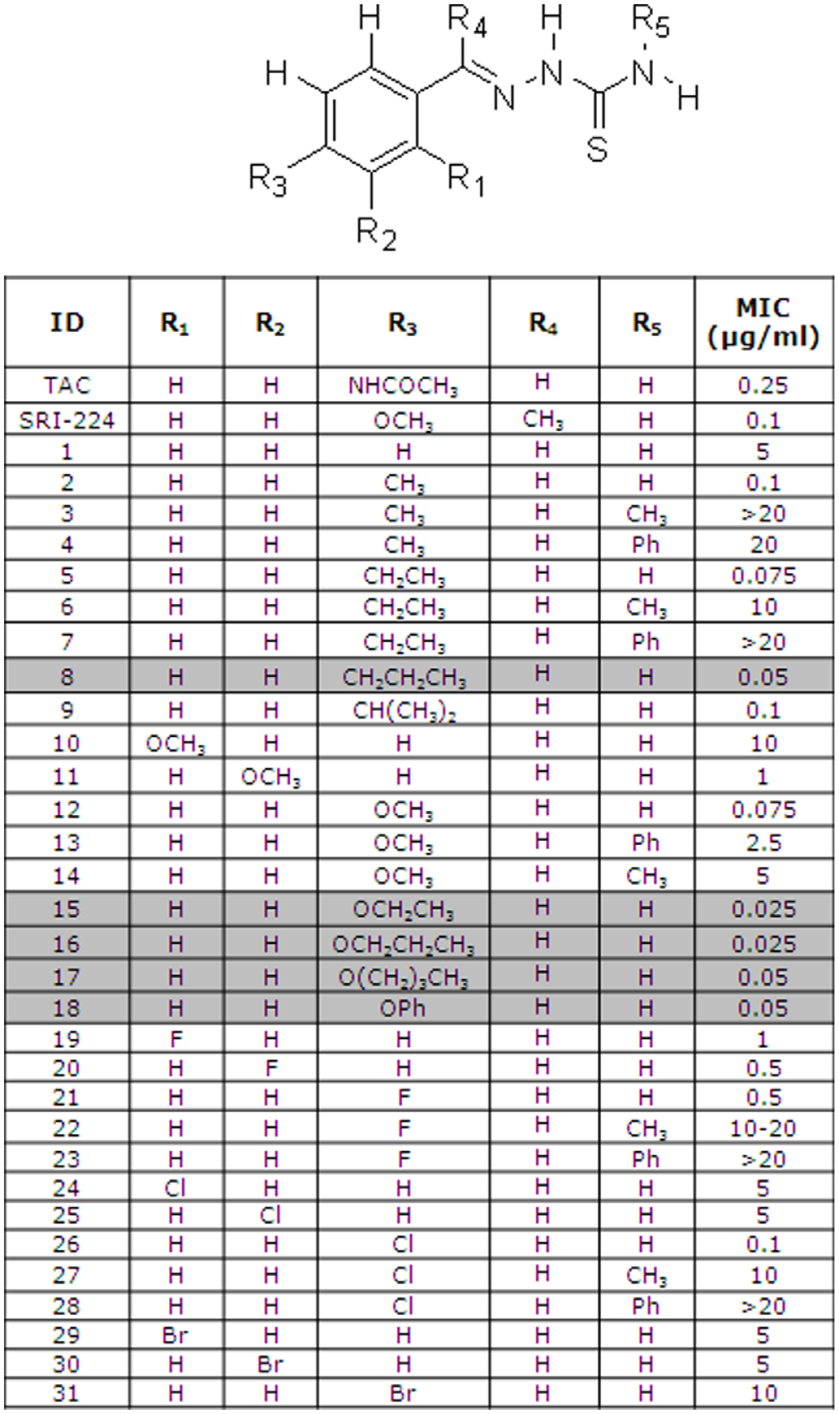
Structures of chemical analogues of thiacetazone and their corresponding minimum inhibitory concentrations in *M. tuberculosis*. Shaded lanes highlight the more efficient analogues.

Due to the low cost of TAC, it was widely used in combination with INH as a first-line antitubercular therapy in poor countries [24] and has also been used throughout the developing world [24] to treat patients infected with multi-drug resistant *M. tuberculosis* strains [24], [31], [32]. However, secondary toxic effects, especially severe skin reactions in patients co-infected with HIV, led to its removal from the antitubercular chemotherapeutic portfolio [33], [34]. We reasoned that if new TAC-related compounds could be identified that have decreased MICs and less severe side-effects, they could be administered at lower doses and consequently induce fewer or no side-effects, and thus represent a valuable alternative for the treatment of drug-resistant strains. Preclinical pharmacokinetics have identified one such TAC analogue (KBF611) that is considered suitable for further clinical evaluation [35]. TAC was therefore chosen as a target pharmacophore for the preparation of analogues. Having observed that the simple modification of the parent TAC scaffold to create SRI-224 led to a greater than two-fold increase in potency, we investigated the effect of further simple structural modifications. To achieve this task we prepared a modest library of 31 compounds using a standard protocol for the generation of imines *via* the reaction of thiosemicarbazide, a derivative thereof, and a range of mono-substituted aromatic aldehydes. All these new TAC-related compounds were evaluated for their ability to inhibit growth of *M. tuberculosis* mc^2^7000 by determining their respective MICs. As shown in Figure 3, the compounds exhibited a large range of activity, from very active compounds to analogues exhibiting only modest or poor activity. In particular, compounds **8**, **15**, **16**, **17** and **18** were found to be 5- to 10-fold more active than the parental TAC molecule. This extends our previous studies and confirms that newer, more effective analogues might be developed for clinical use [20]. The studies described below involve two of the most effective agents synthesized, **15** and **16**, but larger numbers of analogues will be required to develop a robust structure-activity relationship for the thiosemicarbazone class of antitubercular agents.

### Structure-activity and Further Considerations for the Development of the TAC Scaffold

Modification of the aromatic 4-position to give structural analogues directly related to TAC revealed that O-ethyl **15** and O-propyl **16** groups were able to increase the potency by 10 fold, with MIC values of 0.025 µg/ml (Fig. 3). The corresponding ethyl **5** and propyl **8** groups appeared to have result in a smaller increase in potency, with MIC values of 0.075 µg/ml and 0.5 µg/ml, respectively, and shorter **2, 3** or bulkier **9** substituents resulted in even lower potency. Substitution with halogens appeared not to increase potency although the size of the halogen seemed to significantly affect the activity of the compounds. When modified with an F **21** at the 4-position, the MIC was 0.5 µg/ml, but the MIC decreased when replaced by Cl **26** and increased dramatically with Br **31**, yielding MIC values of 0.1 µg/ml and 10 µg/ml, respectively. Substitution of the aromatic ring at the 2- and 3- positions resulted in lower potency than the corresponding 4-position analogues, with the only exception the 4-Br analogue **31,** which was only half as active as analogues **29** and **30**. Interestingly, modification of the carbothiamide section of the molecule at R_5_ with either methyl or phenyl dramatically lowered activity in all cases.

In conclusion, the structure-activity relationships for our set of compounds indicated that: 1) modification at the 4-position gave the best activity when a balance of size and flexibility was achieved with O-propyl and O-ethyl substituents; 2) monosubstitution of the ring at other positions was not favourable; and 3) modification of the carbothiamide section was not tolerated.

### TAC Analogues 15 and 16 Inhibit *M. tuberculosis* Mycolic Acid Biosynthesis

**Figure 4:**
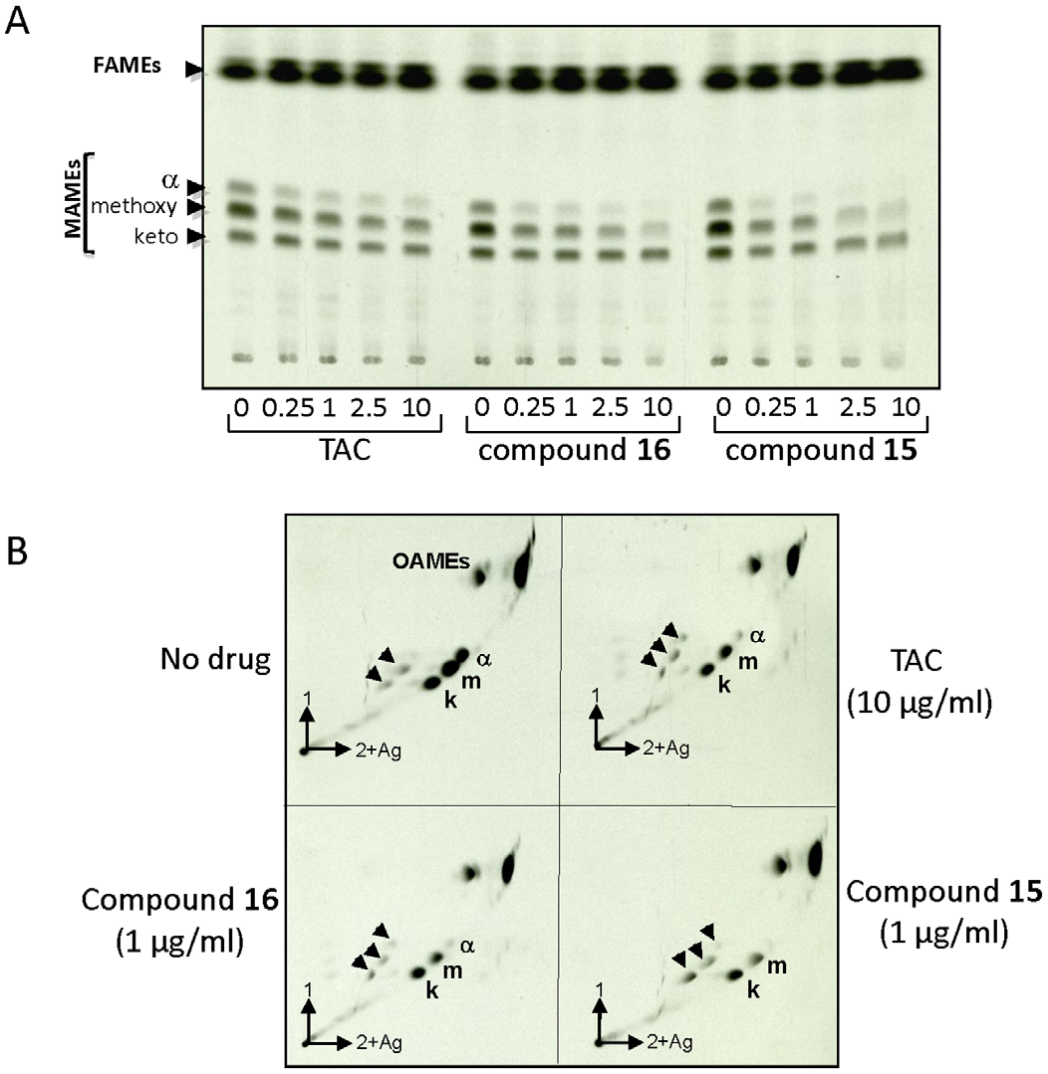
Dose-response effects of TAC and related analogues on mycolic acid biosynthesis in *M. tuberculosis.* The inhibitory effect on the incorporation of [2-^14^C]acetate was assayed by labeling in the presence of increasing concentrations of TAC, **15 **or **16**. The corresponding fatty acid methyl esters (FAME) and mycolic acid methyl esters (MAME) were extracted and equal counts were loaded onto a TLC plate. (**A**) **1D TLC profile of MAMEs.** Radiolabeled lipids (40,000 cpm) were resolved with hexane/ethyl acetate (19/1, v/v, 2 runs) and exposed overnight to a film. (**B**) **2D TLC profile of MAMEs.** Radiolabeled lipids (30, 000 cpm) were resolved with hexane/ethyl acetate (19/1, v/v, 2 runs) in the first dimension and petroleum ether/diethyl ether (17/3, v/v, 3 runs) in the second direction on 10% silver nitrate-impregnated plates and exposed for two days to a film. Α, α-mycolates; keto, ketomycolates, methoxy, methoxymycolates. Arrowheads indicate positions of the unsaturated mycolic acid species. OAME, oleic acid methyl ester.

*M. tuberculosis* carries three types of mycolic acids: di-cyclopropanated α-mycolic acids and oxygenated, methoxy- and keto-mycolic acids. Synthesis of cell wall lipids, particularly mycolic acids, is essential for survival of mycobacteria *in vivo*. To evaluate the effect of TAC analogues on mycolic acid biosynthesis, we extracted methyl mycolic acids and fatty acid methyl esters from cultures grown with increasing concentrations of TAC, **15 **or **16**, followed by metabolic labeling with [^14^C]acetate and TLC/autoradiography analysis. In agreement with previous reports [17], 20,23,24, TAC alters the synthesis of mycolates, especially of α-mycolic acids (Fig. 4A), while the synthesis of methoxy-mycolic acids and keto-mycolic acids appeared more resistant to TAC inhibition, at least at the concentrations tested [24]. This general effect of TAC on mycolic acid biosynthesis is particularly relevant to our recent observation that exposure of mycobacteria to TAC is associated with a decreased production of a cell wall arabinoglycerolipid, designated DMAG, for dimycolyl diarabinoglycerol [36]. Furthermore, inhibition of DMAG production by TAC was dependent on the presence of a functional MmaA4 [36]. From these data, it can be inferred that the disappearance of DMAG following treatment with TAC is a direct consequence of the inhibition of mycolic acid synthesis.

Analogues **15** and **16** were also found to inhibit α- and methoxy-mycolic acid synthesis in a dose-dependent manner with almost complete abrogation of α-mycolic acid biosynthesis at 10 µg/ml (Fig. 4A). Furthermore, a more thorough comparative analysis of the mycolic acid profile resolved by two-dimensional silver nitrate-impregnated TLC plates (Fig. 4B) showed that the inhibition of mycolic acid synthesis was accompanied by a modest, but reproducible, accumulation of unsaturated mycolic acids, presumably due to the inhibition of SAM-dependent methyltransferases involved in introducing cyclopropane rings on the meromycolates, as reported previously in other mycobacterial strains [20]. While this effect was very limited following TAC treatment, accumulation of unsaturated keto-mycolic acid precursors was more pronounced after exposure to higher concentrations of **15** and **16** (data not shown), consistent with our previous observations using the SRI-224 analogue [20].

One would expect that all mycolic acid subspecies would be inhibited concomitantly, leading to complete cessation in mycolic acid biosynthesis, as is seen with specific FAS-II inhibitors such as INH or ETH. FAS-II components are all essential and known to participate in the meromycolyl-ACP elongation steps, and it is during this process that the specific methyltransferases involved in the formation of the meromycolic acid are likely to operate. That keto-mycolic acids are refractory to TAC inhibition is somehow intriguing and argues against the HadABC being directly targeted by TAC. Supporting this view, both the recent study by Belardinelli and Morbidoni [24] and the present study failed to identify mutations within the HadB subunit bearing the catalytic activity. Mutations only occurred in the HadA and HadC subunits, perhaps stabilizing the complex and presenting the growing acyl-ACP substrate to HadB [37], thought to be the enzymatically active component of the complex. Elegant work demonstrated that the FAS-II system of *M. tuberculosis* is organized in specialized interconnected complexes composed of the condensing enzymes, dehydratase heterodimers and the methyltransferases [38], [39], [40]. This led the authors to propose that because the interactions amongst these enzymes are crucial and their disruption detrimental for *M. tuberculosis* survival, the protein interactions could represent attractive drug targets. Whether this is the case for TAC remains to be established experimentally but seems highly conceivable. Our results also emphasize the possibility that EthA-activated TAC and related analogues are likely to inhibit mycolic acid biosynthesis by physically altering the interactions of HadAB and HadBC dehydratases with other FAS-II components, particularly those involved in the formation of the meromycolic acid. The disruption of the interactions between Had complexes and SAM-dependent methyl transferases (CMAS) would explain the mycolic acid profile of **15**- and **16**-treated mycobacteria: less mycolates (as a result of Had alteration) and accumulation of the unsaturated precursors (as a result of CMAS inhibition). Why **15** and **16** are more active than TAC remains unknown, but they could be more stable and/or more efficient perturbagenic molecules than TAC. The ability of **15** to inhibit keto-mycolic acid biosynthesis could be explained by a greater capacity to disrupt specific interactions between Had complexes and the still unknown putative redox enzyme that converts the hydroxymycolate products of MmaA4 into keto-mycolic acids. While these models are attractive, confirmation will require further study and more information on the structure of the proteins, their mode of interaction and how the drugs alter these interactions.

### Overexpression of the Had Heterocomplexes Lead to High Resistance Levels to TAC Analogues

The above results prompted us to investigate whether overexpression of the FAS-II dehydratase would result in resistance to the TAC analogues. The different HadAB, HadBC or HadABC heterocomplexes were overproduced in *M. tuberculosis* by ligating the corresponding genes into the pMK1 or pVV16 vectors, in which the genes of interest are under the control of the constitutive *hsp60* promoter and carry either an N-terminal (pMK1) or a C-terminal (pVV16) His-tag [28]. As previously observed [24], overexpression of *had*AB, *had*BC or *had*ABC led to high-level resistance to TAC (Table 3). The MICs for *hadAB* were 10-fold greater than those of the bacteria carrying the plasmid vector, while the MICs for bacteria containing the *hadBC* and *hadABC* plasmids were more than 200-fold greater. Similarly, overproduction of the different heterocomplexes induced high resistance levels to **15** and **16**, with MICs that were 10-fold (**15** against a strain with the *hadAB*) to 200-fold greater than the MICs of strains with the vector alone (Table 3). These data suggest that the HadAB and HadBC heterocomplexes differ in their ability to generate resistance to the **15** and **16** analogues, which may reflect differences in their interactions with the target proteins. The nature of the modification at 4-position of the aromatic ring (O-ethyl in **15** versus O-propyl in **16**) appears critical in defining the resistance level of the various Had-overproducing strains. This observation is also supported by the fact that compound **17**, that possesses an additional methyl group compared to **16** (O-butyl at 4-position) exhibits activity levels comparable to **15** against the Had-overproducing strains (Table 3).

**Table 3:** MIC of TAC and its related analogues **15**, **16** and **17** against recombinant *M. tuberculosis* strains overexpresssing the different dehydratase complexes^a^.

compound strain	TAC	15	16	17
**pMK1**	0.25	0.025	0.05	0.05
**pMK1_** ***hadAB***	2.5	0.25	2.5	0.25
**pVV16**	0.25	0.025	0.025	0.05
**pVV16_** ***hadBC***	>50	1	>10	1
**pMV261_** ***hadABC***	>50	1	>10	1

a MIC_99_ expressed in µg/ml was determined by dilution on solid agar medium 7H10 supplemented with OADC.

In conclusion, TAC is generally dismissed as a very toxic drug, but this work suggests that more active analogues with reduced MIC levels might be effectively administered at significantly lower doses. While investigating the possible toxic effects of the new analogues is beyond the scope of this study, the results show that it is possible to synthesize several more potent analogues, raising the hope that one can be found for which a lower effective dose will induce a much lower, acceptable level of side effects. It is clear from this study that simple modification of the TAC scaffold with compounds **15** and **16** permits optimisation whilst maintaining the same sites of action, as these compounds also inhibit mycolic acid biosynthesis. While further work is required on the structural modification of this scaffold, our data suggests the possibility of developing a molecule derived from the TAC structure that inhibits mycolic acid biosynthesis with a pharmacokinetic/pharmacodynamic profile that permits lower patient dosing than with TAC and thus potentially reduces or eliminates the toxicity associated with the drug. Before these or other TAC analogues can be seriously considered as potential drugs for the treatment of MDR or XDR TB, pharmacological and toxicological studies are needed to test their toxicity. Additionally, solving the structures of HadA, HadB and HadC would facilitate a better understanding of the mechanism of action of TAC and its analogues and help to precisely define how the mutations described here confer resistance.
